# Comparison of weekly and triweekly cisplatin regimens during concurrent chemoradiotherapy for nasopharyngeal carcinoma

**DOI:** 10.1186/s12885-019-5688-z

**Published:** 2019-05-22

**Authors:** Kailin Wang, Jun Dong, Shasha He, Xia Wang, Chang Jiang, Pili Hu, Jiangui Guo, Xiuyu Cai, Xicheng Wang

**Affiliations:** 1 Department of Oncology, The First Affiliated Hospital of Guangdong Pharmaceutical University, Guangdong Pharmaceutical University, 19 Nonglinxia Road, Guangzhou, 510062 China; 2 Department of Integrated Therapy in Oncology, Sun Yat-sen University Cancer Center, State Key Laboratory of Oncology in South China, Collaborative Innovation Center for Cancer Medicine, Guangdong Key Laboratory of Nasopharyngeal Carcinoma Diagnosis and Therapy, 651 East Dongfeng Road, Guangzhou, 510060 China; 3 Department of Radiation, Sun Yat-sen University Cancer Center; State Key Laboratory of Oncology in South China; Collaborative Innovation Center for Cancer Medicine, Guangdong Key Laboratory of Nasopharyngeal Carcinoma Diagnosis and Therapy, Guangzhou, 510060 China; 4 Department of Radiation Oncology, The First People’s Hospital of Foshan, Foshan, 528000 China

**Keywords:** Nasopharyngeal carcinoma, Cisplatin, Concurrent chemoradiotherapy, Survival

## Abstract

**Background:**

We compared the survival outcomes and acute toxicities of weekly and triweekly cisplatin regimens during concurrent chemoradiotherapy (CCRT) in nasopharyngeal carcinoma (NPC) patients.

**Methods:**

Patients were treated with CCRT alone. CCRT was initiated on the first day of RT. Cisplatin 30–40 mg/m^2^ was infused on days 1, 8, 15, 22, 29, 36 and 43 in the Weekly Group, while cisplatin 80–100 mg/m^2^ was delivered on days 1, 22 and 43 in the Triweekly Group. The survival outcomes were revealed by the Kaplan-Meier method and Cox regression modelling to measure 5-year overall survival (OS), disease-free survival (DFS), locoregional relapse-free survival (LRFS) and distant metastasis-free survival (DMFS).

**Results:**

Ninety-three (28.9%) patients received three to 7 cycles of cisplatin weekly (Weekly Group) and 229 (71.1%) patients received two to 3 cycles of cisplatin triweekly (Triweekly Group). Five-year OS (weekly vs. triweekly, 96.7% vs. 88.3%, *P* = 0.036) and DFS (weekly vs. triweekly, 90.7% vs. 80.5%, *P* = 0.028) were better in the Weekly Group than in the Triweekly Group. The weekly vs. triweekly 5-year DMFS and LRFS rates were: DMFS, 96.7% vs. 91.4%, χ^2^ = 2.694, *P* = 0.101; LRFS, 96.3% vs. 93.5%, χ^2^ = 1.317, *P* = 0.251. Cisplatin delivery regimen was not an independent prognostic factor. The incidence rate of acute toxicities was similar between the groups.

**Conclusions:**

Compared with Triweekly cisplatin regimen, Weekly regimen may be a better choice during CCRT.

## Background

Nasopharyngeal carcinoma (NPC) is a rare form of cancer, and is well known for its high mortality and morbidity in certain ethnic and regional populations, especially Southeast-Asian individuals. The incidence and mortality rate of NPC are about 2 to 3 times higher in men than in women [1], and most patients with the condition exhibit locoregional or distant metastases at diagnosis. Advances in treatment technology and medicine have led to clear improvements in the local control and overall survival (OS) rates of NPC. Because of the radio- and chemo-sensitivity of the condition, patients receive systemic therapy based on concurrent chemoradiotherapy (CCRT). Even for locoregionally advanced NPC, good local control of diseases can be achieved by using this therapy [2, 3]. Gradually, intensity-modulated radiation therapy (IMRT) has become the preferred mode of radiotherapy (RT) delivery because of its low incidence of severe complications.

Several previous studies have demonstrated the survival benefits of CCRT relative to RT alone [4–9]. Triweekly cisplatin (100 mg/m^2^ infusion on Days 1, 22, and 43) is commonly used in these trials. However, compliance with this intensive regimen is poor because of the high incidence of acute toxicity [7, 9, 10]. To reduce acute toxicity, a weekly cisplatin regimen (40 mg/m^2^ infusion on Days 1, 8, 15, 22, 29, 36, and 43) was developed for use during CCRT [8, 11]. A recent report showed that smaller cisplatin doses administered more frequently during CCRT for head and neck cancers can result in a better tolerance of acute toxicities without compromising efficacy [12].

Although several investigations have compared the efficacies of weekly and triweekly cisplatin regimens during CCRT, adjuvant chemotherapy (AC) was performed after CCRT in these studies [13–15]. There are no head-to-head comparisons of weekly and triweekly cisplatin regimens in patients who received CCRT alone. In addition, there are no guidelines that definitively indicate which regimen is superior. Therefore, the purpose of this retrospective research was to compare the long-term survival outcomes and acute toxicities of weekly and triweekly cisplatin regimens in NPC patients who received CCRT alone.

## Methods

This study involved retrospective analysis of 322 newly diagnosed and non-metastatic NPC patients, which treated by CCRT alone at the Sun Yat-sen University Cancer Center, Guangzhou, Guangdong, China, from January 2010 to November 2013. All patients were restaged on basis of the eighth edition of the American Joint Committee on Cancer (AJCC) staging system [16]. Histological subtypes of nasopharyngeal carcinoma were classified according to the WHO tumor classification rules, they are: keratinizing squamous-cell carcinoma (type I), differentiated non-keratinous carcinoma (type II), undifferentiated non-keratinous carcinoma (type III).

The procedures in the various studies involving human participants are consistent with the ethical standards established by national research commissions or institutions, as is the case with the Helsinki declaration and its subsequent revisions. The authenticity of this paper was verified by uploading the original data to the public platform of Research Data Deposit (www.researchdata.org.cn) and obtaining the approval number of RDD (RDDA2018000818).

### Enrollment criteria

The principal eligibility criteria were:

- Initial treatment and no metastasis at diagnosis;
- treatment with only IMRT and cisplatin as the sole chemotherapy drug;
- adequate hematologic, hepatic, and renal functions;
- The concept of Stage-I–IVA disease defined by the eighth edition of the AJCC staging system [16].

The exclusion criteria were:

- History of prior malignancy or previous treatment for NPC;
- The existence of uncontrolled life-threatening diseases;
- Treatment with neoadjuvant or adjuvant chemotherapy;
- Radiotherapy techniques other than IMRT.

### Treatment

All patients received the treatment of CCRT alone. CCRT was started on the first day of RT. The choice of weekly or triweekly cisplatin regimen was based on an evaluation of each patient’s situation and aspirations by oncologists. Before thermotherapy with cisplatin, hydration was necessary for the Triweekly Group; dexamethasone plus 5-hydroxytryptamine type 3 antagonists were routinely used as antiemetic prophylaxis for all groups.

In the Weekly Group, 30–40 mg/m2 of cisplatin was infused on Days 1, 8, 15, 22, 29, 36, and 43. In the Triweekly Group, 80–100 mg/m2 of cisplatin was delivered on Days 1, 22, and 43.

IMRT was performed throughout CCRT. The prescribed cumulative radiation doses were 66–72 Gy/30–33 fraction for the gross tumor volume of the nasopharynx, the doses of positive cervical lymph nodes were 64–70 Gy, 60–63 Gy and 54–56 Gy for the high- and low-risk clinical target volume, respectively. RT took 6–7 weeks. Due to the limitation of dose tolerance mentioned in Radiation Therapy Oncology Group 0225 protocol, it is necessary to reduce the use of measurement as far as possible to avoid sacrificing the coverage of tumor target [17].

### Follow-up

During the first 2 years after treatment, follow-up assessment was conducted at least every 3 months, every 6 months for the next three to 5 years, and annually thereafter until death. Every follow-up contained physical examination, nasopharynx and neck magnetic resonance imaging, nasopharyngoscopy, thoracic computed tomography, abdominal ultrasound or computed tomography, bone scan, and some hematological parameters, especially Epstein-Barr virus (EBV) DNA load.

### Survival outcome evaluation

Follow-up was calculated from the date of diagnosis to the date of last follow-up or death. Overall survival (OS) was regarded as the time from first diagnosis to death, or the last follow-up. Disease-free survival (DFS) was regarded as the time from diagnosis to death, disease progression at locoregional and/or distant sites, or the last follow-up. Distant metastasis-free survival (DMFS) was regarded as the time from diagnosis to the first time observation of distant metastases. Locoregional relapse-free survival (LRFS) was regarded as the time from diagnosis to the relapse.

### Toxicity evaluation

An assessment of toxicity was performed before and after each round of chemotherapy. The most serious toxic events that occurred during CCRT were counted. Acute toxicities were scored on the basis of the *Common Terminology Criteria for Adverse Events* version 4.03. [18]. Grade 3–4 adverse events (as severe events of acute toxicities) that occurred during CCRT were counted.

### Statistical analysis

We used a chi-square test or Fisher’s exact test to compare the clinical characteristics and acute toxicity rates of the two treatment groups. We used Student’s *t*-test to compare the cumulative dose of cisplatin in each group. Using the Cox proportional hazards regression model to analysis the clinical variables, and variables with *P*-values < 0.05 were used in the following multivariate analyses. Survival was measured by the Kaplan-Meier, and the differences between curves were analyzed by the log-rank test. Statistical analysis was performed using SPSS version 19.0 (IBM Corp, Armonk, NY, USA). Each statistical test was two-sided; *P* < 0.05 was deemed to have statistical significance [19].

## Results

### Baseline characteristics

Registered a total of 322 eligible patients, of them, 93 (28.9%) patients required weekly cisplatin treatment (Weekly Group) and 229 (71.1%) received triweekly cisplatin (Triweekly Group) during CCRT. The median age among the whole cohort was 44 years old. The plasma pre-treatment EBV DNA load cutoff value of 4000 copies/ml was based on a previous study, where the use of this value led to significant risk stratification [20]. Sixty-two (66.7%) patients in the Weekly Group and 146 (63.8%) in the Triweekly Group had a plasma EBV DNA load ≤4000 copies/ml, and 31 (33.3%) patients in the Weekly Group and 83 (36.2%) in the Triweekly Group had a plasma EBV DNA load higher than the cutoff value. The Weekly and Triweekly Group means of cumulative cisplatin were 190. 54 mg and 202.97 mg, respectively (*P* = 0.062). Most of the patients were non-smokers, non-drinkers, suffered from non-keratinizing undifferentiated carcinoma (type III), and were diagnosed with Stage-III–IVA disease. There were no significant differences in baseline demographics and disease characteristics between the two groups. Details are shown in Table 1.

**Table 1:** Baseline characteristics of the 322 patients who received concurrent chemoradiotherapy

Characteristic	Weekly Group		Triweekly Group		Total		*P*
	No.	%	No.	%	No.	%	
Age							0.985^b^
≤44	47	50.5	116	50.7	163	50.6	
>44	46	49.5	113	49.3	159	49.4	
Sex							0.076^b^
Male	83	89.2	217	94.8	300	93.2	
Female	10	10.8	12	5.2	22	6.8	
Smoking							0.915^b^
No	74	79.6	181	79.0	255	79.2	
Yes	19	20.4	48	21.0	67	20.8	
Drink							0.201^b^
No	91	97.8	228	99.6	319	99.1	
Yes	2	2.2	1	0.4	3	0.9	
Histology^a^							0.560^b^
Non-keratinizing Undifferentiated (type III)	93	100	226	98.7	319	99.1	
Others (type I and II)	0	0	3	1.3	3	0.9	
T stage (8^th^ AJCC)							0.941^b^
T1–2	39	41.9	95	41.5	134	41.6	
T3–4	54	58.1	134	58.5	188	58.4	
N stage (8^th^ AJCC)							0.172^b^
N0–1	80	86.0	182	79.5	262	81.4	
N2–3	13	14.0	47	20.5	60	18.6	
Stage (8^th^ AJCC)							0.475^b^
I–II	36	38.7	79	34.5	115	35.7	
III–IVA	57	61.3	150	65.5	207	64.3	
EBV							0.621^b^
≤4000 copy/ml	62	66.7	146	63.8	208	64.6	
>4000 copy/ml	31	33.3	83	36.2	114	35.4	
Cumulative cisplatin	190.54	53.27^d^	202.97	54.39^d^			0.062^c^

*Abbreviations*: *T* NPC tumor stage, *N* nodal stage, *CCRT* concurrent chemoradiotherapy, *AJCC* American Joint Committee on Cancer, *EBV* Epstein–Barr virus

^a^Histology was categorized according to the WHO Classification of Tumors

^b^Chi-square test or Fisher’s exact test

^c^Student’s *t*-test

^d^Standard deviation

### Survival outcomes

**Fig. 1:**
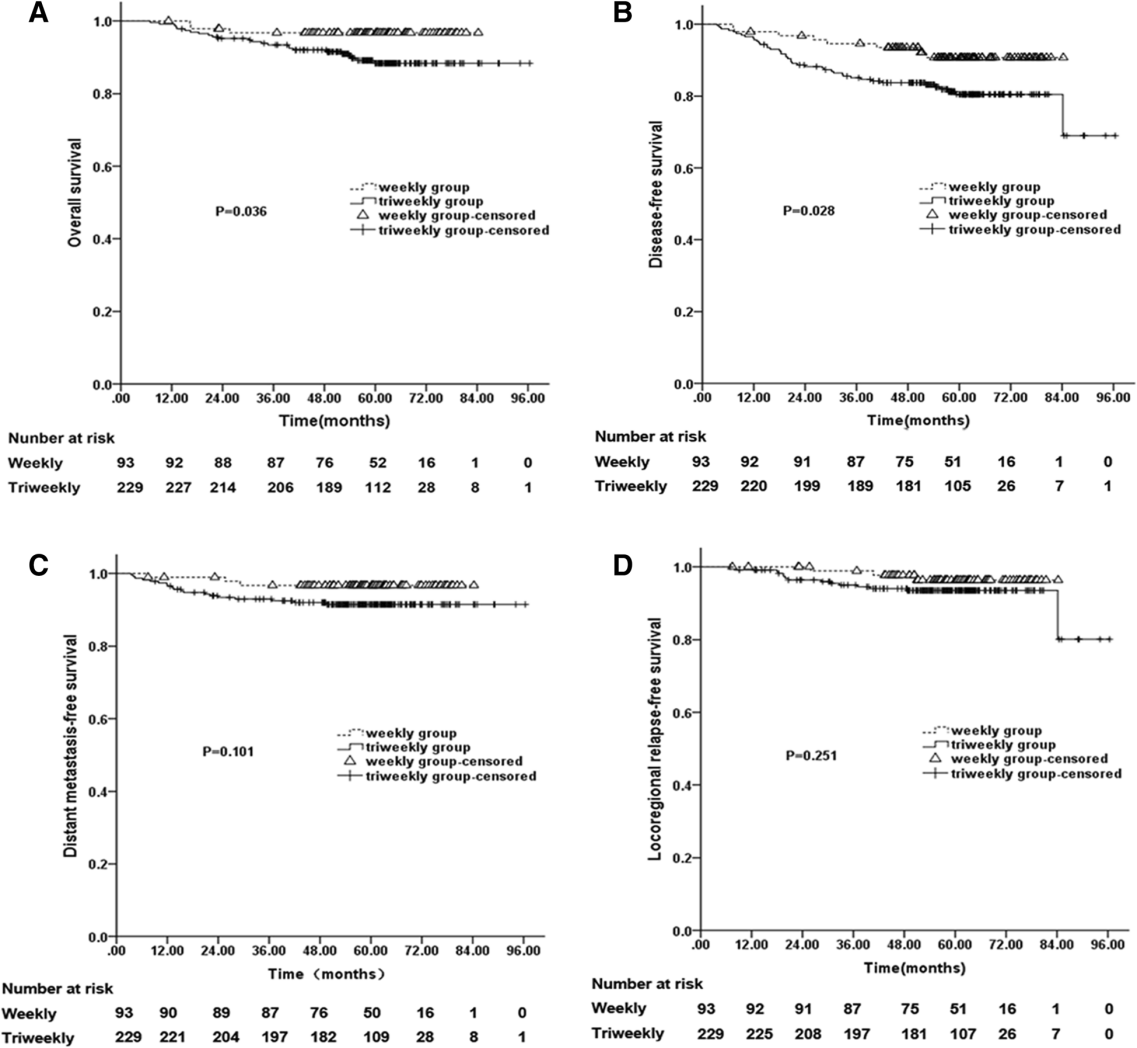
Survival curves for patients with NPC. The overall survival (OS) curves showed that the Weekly group was better than Triweekly group (*P* = 0.036, there was a significant difference between the groups) (**a**). The disease-free survival (DFS) curves showed that the Weekly group was better than the Triweekly group (*P* = 0.028, there was a significant difference between the groups) (**b**). The distant metastasis-free survival (DMFS) curves (**c**) and the locoregional relapse-free survival (LRFS) curves (**d**) revealed no significant difference between the two groups

The median follow-up time for the entire cohort was 60.2 months (range, 7–96.4 months). The overall 5-year OS, DFS, DMFS, and LRFS rates were 90.8, 83.8, 92.9, and 94.3%, respectively. The 5-year OS rate of the Weekly Group was better than that of the Triweekly Group (Fig. 1a). The log-rank test revealed that there was a significant difference in 5-year OS between the groups (weekly vs. triweekly, 96.7% vs. 88.3%, χ2 = 4.382, *P* = 0.036). The 5-year DFS rates of the Weekly and Triweekly Groups were 90.7 and 80.5%, respectively, and exhibited a significant difference between the groups (χ2 = 4.831, *P* = 0.028; Fig. 1b). Regarding the weekly vs. triweekly 5-year DMFS and LRFS rates, there were no significant differences between the two groups (DMFS, 96.7% vs. 91.4%, χ2 = 2.694, *P* = 0.101; LRFS, 96.3% vs. 93.5%, χ2 = 1.317, *P* = 0.251; Fig. 1c and d).

### Acute toxicities

There was a similarity in the incidence of grade 3–4 treatment-related adverse events, such as hematologic and non-hematologic toxicity events, between the Weekly and Triweekly Groups. Hematologic toxic events were more frequent in the Weekly Group than in the Triweekly Group. Seven (7.5%) patients in the Weekly Group and eight (3.5%) in the Triweekly Group exhibited Grade 3–4 thrombocytopenia. However, there was no significant difference in the incidence of Grade 3–4 thrombocytopenia between the groups (*P* = 0.12). Two (2.2%) patients in the Weekly Group and one (0.4%) in the Triweekly Group developed Grade 3–4 anemia, and 21 (22.6%) patients in the Weekly Group and 38 (16.6%) in the Triweekly Group developed Grade 3–4 leucopenia. There were no significant differences in the incidences of Grade 3–4 anemia (*P* = 0.147) and Grade 3–4 leucopenia (*P* = 0.208) between the groups. The incidence of Grade 3–4 non-hematologic toxicities, including in the mucosa, skin, nausea-vomiting, creatinine-increased and ototoxicity were similar between the groups. There were no deaths due to treatment. The toxicities after the completion of CCRT in each group are listed in Table 2.

**Table 2:** Treatment related toxicities

Toxicities	Weekly		Triweekly		P*
	No.	%	No.	%	
Hematologic					
Thrombocytopenia					0.120
<G3	86	92.5	221	96.5	
≥G3	7	7.5	8	3.5	
Anemia					0.147
<G3	91	97.8	228	99.6	
≥G3	2	2.2	1	0.4	
Leucopenia					0.208
<G3	72	77.4	191	83.4	
≥G3	21	22.6	38	16.6	
Non-hematologic					
Mucosa					0.170
<G3	89	95.7	209	91.3	
≥G3	4	4.3	20	8.7	
Skin					1.000
<G3	92	98.9	227	99.1	
≥G3	1	1.1	2	0.9	
Nausea-vomiting					0.786
<G3	89	95.7	216	94.3	
≥G3	4	4.3	13	5.7	
Creatinine-increased					0.289
<G3	92	98.9	229	100.0	
≥G3	1	1.1	0	0.0	
Ototoxicity					1.000
<G3	93	100.0	227	99.1	
≥G3	0	0.0	2	0.9	

*Abbreviations*: *G* grade

*Chi-square test or Fisher’s exact test

### Univariate and multivariate analyses

Pre-treatment EBV DNA load (≤4000 copies/ml vs. > 4000 copies/ml, hazard ratio [HR] = 3.402, 95% confidence interval [CI] = 1.525–7.589, *P* = 0.003) was an independent prognostic factor for OS. The cisplatin delivery regimen (weekly vs. triweekly, HR = 2.795, 95% CI = 0.836–9.35, *P* = 0.095) was not an independent prognostic factor for OS. Regarding DFS, pre-treatment EBV DNA load (≤4000 copies/ml vs. > 4000 copies/ml, HR = 2.365, 95% CI = 1.355–4.126, *P* = 0.002) and node (N) classification (N0–1 vs. N2–3, HR = 2.18, 95% CI = 1.157–4.109, *P* = 0.016) were independent prognostic indicators. The regimen of cisplatin delivery (weekly vs. triweekly, HR = 2.071, 95% CI = 0.969–4.426, *P* = 0.06) was not an independent prognostic factor for DFS (Table 3). Regarding LRFS (weekly vs. triweekly, HR = 2.665, 95% CI = 0.788–9.005, *P* = 0.115) and DMFS (weekly vs. triweekly, HR = 1.985, 95% CI = 0.57–6.908, *P* = 0.281) in univariate analyses, the cisplatin delivery regimen was ineligible for inclusion in the multivariate analysis.

**Table 3:** Multivariate analyses in the overall population

Variable	HR	95% CI	P*
OS			
EBV	3.402	1.525–7.589	0.003
Cisplatin regimen	2.795	0.836–9.350	0.095
DFS			
EBV	2.365	1.355–4.126	0.002
N stage	2.180	1.157–4.109	0.016
Cisplatin regimen	2.071	0.969–4.426	0.060

*Abbreviations*: *OS* overall survival, *EBV* Epstein–Barr virus, *DFS* disease-free survival, *CI* confidence interval, *HR* hazard ratio

*Chi-square test or Fisher’s exact test

### Subgroup analyses

**Fig. 2:**
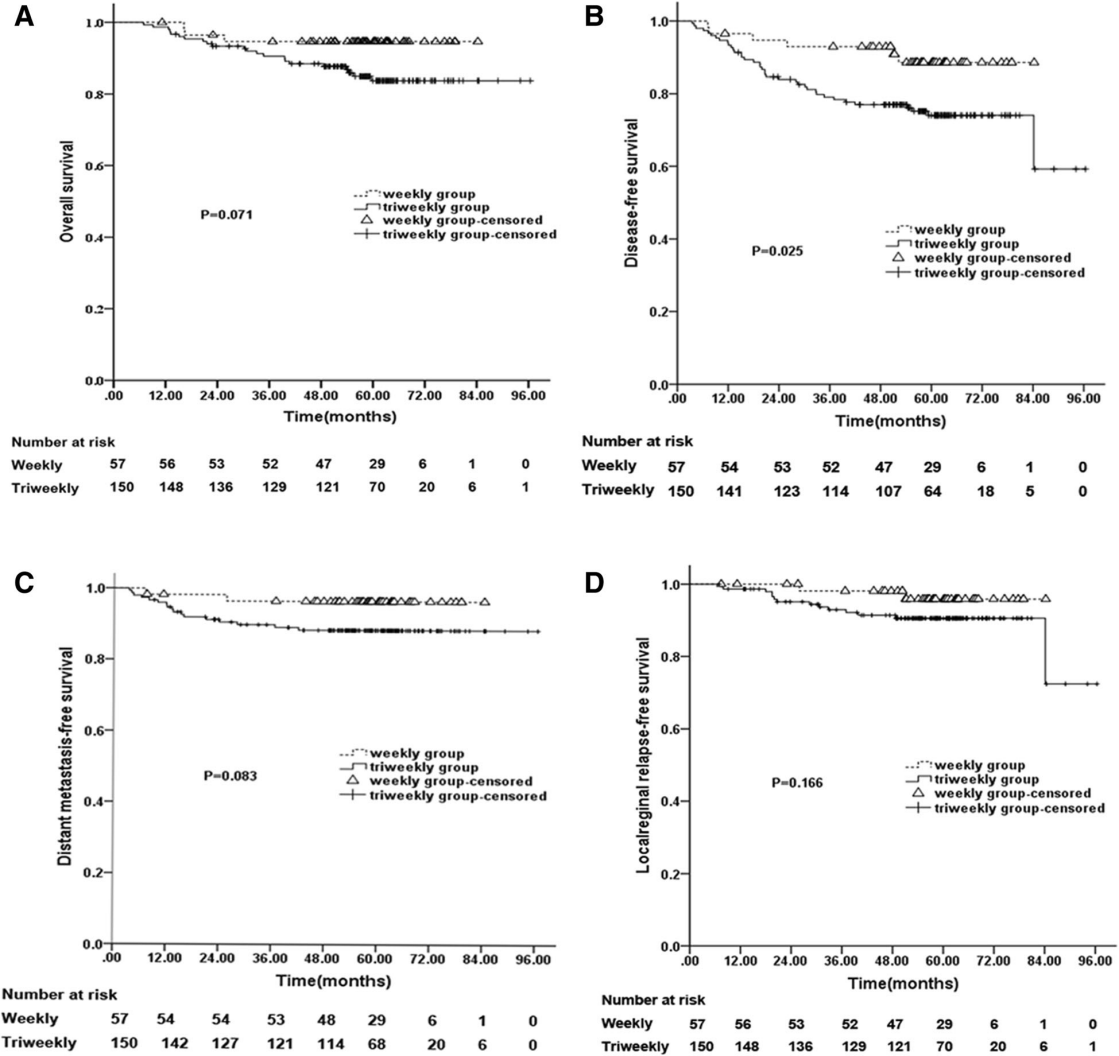
Survival curves for patients with Stage-III–IVA NPC. The OS curves showed there was no significant difference between the two groups (**a**). The DFS curves showed that the Weekly group was better than the Triweekly group (*P* = 0.025, there was a significant difference between the groups) (**b**). The DMFS (**c**) and LRFS (**d**) curves showed no significant differences between the two groups

In the entire cohort, 207 (64.3%) patients had Stage-III–IVA disease, including 57 (61.3%) patients in the Weekly Group and 150 (65.5%) in the Triweekly Group. The 5-year DFS of Stage-III–IVA patients in the Weekly Group was better than that of Stage-III–IVA patients in the Triweekly Group (weekly vs. triweekly, 88.5% vs. 74%, χ^2^ = 4.991, *P* = 0.025; Fig. 2b), which was statistically significant. The weekly and triweekly 5-year OS rates were 94.6 and 83.7% (χ^2^ = 3.259, *P* = 0.071) (Fig. 2a), the weekly and triweekly 5-year DMFS rates were 96.4 and 88.3% (χ^2^ = 3.004, *P* = 0.083 Fig. 2c), and the weekly and triweekly 5-year LRFS rates were 95.9 and 90.6% (χ^2^ = 1.923, *P* = 0.166 Fig. 2d). The log-rank test revealed that there were no significant differences in the 5-year DMFS and 5-year LRFS rates between the groups (Fig. 2). For patients with Stage-I–II disease, the weekly vs. triweekly survival end-points were as follows: 5-year OS, 100% vs. 97.1% (χ^2^ = 0.875, *P* = 0.35); 5-year DMFS, 97.1% vs. 97.2% (χ^2^ = 0.008, *P* = 0.929); 5-year LRFS, 97.1% vs. 98.7% (χ^2^ = 0.303, *P* = 0.582); and 5-year DFS, 94.3% vs. 92.6% (χ^2^ = 0.025, *P* = 0.874). The log-rank test revealed that there were no significant differences in survival outcomes.

## Discussion

We noticed that the Weekly and Triweekly Groups had similar 5-year DMFS and LRFS rates in this retrospective study. Our results are in agreement with previous studies [13–15], which showed that 40 mg/m^2^ of cisplatin weekly and 100 mg/m^2^ of cisplatin triweekly had similar deliverabilities and outcomes. Also, we found that the 5-year OS and DFS rates of the Weekly Group were better than those of the Triweekly Group, and that the 5-year DFS of patients with Stage-III–IVA disease was better in the Weekly Group than in the Triweekly Group. These results differ from those of previous studies [13–15]. Of course, the patients selected by us differed from those selected in previous studies. All the patients in the studies by Jagdis et al. and Lee et al. [13, 14] and some of the patients in the study by Tao et al. [15] received CCRT plus AC with cisplatin and 5-fluorouracil, whereas our patients received CCRT alone. Furthermore, a small proportion of patients in these three earlier studies received three-dimensional conformal radiotherapy (3D-CRT), but in our study, all patients received IMRT. Besides, previous reports have shown that DFS is better following IMRT than following 3D-CRT [21, 22]. In our study, the survival outcomes were obviously improved compared with those of previous trials [4, 6, 10], which may have been the result of improvements in RT technology, advances in treatment after relapse, and the application of targeted therapy [23–27].

Traditionally, it was thought that more frequent use of smaller doses of cisplatin would produce a lower toxicity profile while maintaining efficacy. This is consistent with the findings of Rampino et al. [12]. Notably, the incidence of Grade 3–4 toxic events was not lower among patients in the Weekly Group than among patients in the Triweekly Group in this study, which is in accordance with the conclusions of Ho et al. [28]. Grade 3–4 toxic events were more frequent in the Triweekly Group (53.3%) than in the Weekly Group (40%), but without statistical significance, in a study by Uygun et al. [29]. In contrast, the KCSG-HN10–02 trial found that the weekly cisplatin regimen caused a higher incidence of severe thrombocytopenia than the triweekly regimen (7.5% vs. 1.8%, *P* = 0.198) [14]. Tao et al. showed that Grade 3–4 hematologic toxic events were more common in the Weekly Group than in the Triweekly Group, but without statistical significance [15]. In this study, more patients in the Weekly Group than in the Triweekly Group exhibited severe hematologic toxic events, including thrombocytopenia (weekly vs. triweekly, 7.5% vs. 3.5%, *P* = 0.12), anemia (weekly vs. triweekly, 2.2% vs. 0.4%, *P* = 0.147), and leucopenia (weekly vs. triweekly, 22.6% vs. 16.6%, *P* = 0.208), but without statistical significance. Every patient was required to undergo assessments of hematologic, renal, and hepatic functions before chemotherapy, but patients in the Weekly Group were evaluated more frequently than patients in the Triweekly Group. This may have delayed the identification of severe advanced events in the Triweekly Group, and may represent an undefined bias regarding toxicity profiles.

The multivariate analysis conclusion that pre-treatment EBV DNA load and N classification are independent prognostic factors has been confirmed by past study [20]. Part of our results differ from those of the KCSG-HN10–02 trial and the studies by Jagdis et al. and Tao et al. [13–15]. We attribute these differences to variations in the study populations, sample sizes, and follow-up durations, and to advances in therapeutic techniques. Canada and Korea do not have high incidences of NPC, meaning that the number of patients is low [13, 14]. Sun Yat-sen University Cancer Center is in south-eastern China, an area with a high prevalence of NPC, and has the highest number of clinical patients with NPC worldwide. In our study, the size of the sample was 322, the patients received treatment with CCRT alone, and the median follow-up time for the entire cohort was 60.2 months (range, 7–96.4 months), beyond which we have continued to follow-up our patients.

This retrospective study has several limitations. First, major limitations, including its retrospective nature, potential selection bias, information bias, and confounding bias, were unavoidable. Second, this study was performed at a single-center located in an area in which NPC is endemic. The main histology of the selected patients was undifferentiated, non-keratinizing carcinoma. Whether our results are applicable to Western countries such as in Europe and North America, where the incidence of NPC is relatively low, remains unclear. Third, our study used simple tumor-nodes-metastasis staging, and did not involve a more detailed subgroup analysis based on patients’ characteristics. Multicenter, prospective, large-scale, randomized controlled trials are necessary to determine the optimal cisplatin regimen during CCRT for the treatment of NPC.

## Conclusions

The incidence of severe acute toxicities was similar between the two groups, whereas the Weekly Group had better performance in OS and DFS than the Triweekly Group. Besides, with the weekly regimen, patients were able to choose outpatient treatment without essential hydration. Thus, the weekly cisplatin regimen may be a better choice during CCRT for the treatment of NPC; prospective randomized controlled clinical trials are needed to confirm this conclusion.

## Abbreviations

3D-CRT: Three-dimensional conformal radiotherapy
AC: Adjuvant chemotherapy
AJCC: American Joint Committee on Cancer
CCRT: Concurrent chemoradiotherapy
CI: Confidence interval
DFS: Disease-free survival
DMFS: Distant metastasis-free survival
EBV: Epstein–Barr virus
HR: Hazard ratio
IMRT: Intensity-modulated radiation therapy
LRFS: Locoregional relapse-free survival
N: Node
NPC: Nasopharyngeal carcinoma
OS: Overall survival
RT: Radiotherapy
